# Method for assessing rodent infestation in plateau based on SegFormer

**DOI:** 10.1371/journal.pone.0325738

**Published:** 2025-06-26

**Authors:** Xiangjie Huang, Guoying Zhang, Chunmei Li, Yaosheng Han, Qing Dong, Hao Wang

**Affiliations:** 1 School of Computer Technology and Application, Qinghai University, Xining, Qinghai, China; 2 Intelligent Computing and Application Laboratory of Qinghai Province, Qinghai University, Xining, Qinghai, China; 3 Joint Logistics Support Force 941th Hospital, People’s Liberation Army, Xining, Qinghai, China; 4 School of Computer and Information Science, Qinghai Institute of Technology, Xining, Qinghai, China; HUN-REN Centre for Ecological Research, HUNGARY

## Abstract

Rodent infestation is a critical factor contributing to grassland degradation, which significantly negatively affects grassland ecosystems. To assess rodent infestation on the plateau, there is an urgent need for a scientifically sound and effective method to detect the distribution of rodent burrows. In response, this study proposes a semantic segmentation approach utilizing the SegFormer model to detect rodent infestation in highland areas. First, we used an unmanned aerial vehicle to collect video data from the plateau and constructed a rodent burrows dataset after processing and precise labeling. Second, to address the issue of SegFormer’s suboptimal performance in segmenting small targets within complex backgrounds and among similar objects, we implemented targeted modifications to enhance its effectiveness for this task. Incorporating the efficient multi-scale attention (EMA) mechanism into SegFormer’s encoder improves the model’s capacity to capture global contextual information. Meanwhile, integrating the multi-kernel convolution feed-forward network (MCFN) into the decoder optimizes the problem of detail recovery and fusion of multi-scale features. We name this method EM-SegFormer (Efficient Multi-scale SegFormer). The experimental results demonstrate that the method achieves relatively good performance on the rodent burrows dataset. This study introduces a novel approach for plateau rodent infestation detection and offers reliable technical support for grassland restoration and management.

## Introduction

The Qinghai–Tibet Plateau is located in the south-central part of Asia, plays a crucial role in China’s water resources and ecological security [1]. This plateau is predominantly covered by grasslands, which account for about 60% of its total area [2]. Qinghai Province is located in the northeastern part of the Qinghai-Tibet Plateau and is one of the most important natural grassland areas in China, as well as a major grazing region [3]. However, due to its remote location and challenging accessibility, obtaining data for environmental monitoring and ecological management in this region has always been difficult. Among the various types of grasslands, the alpine meadows and alpine grasslands constitute the main portion of Qinghai Province’s natural grasslands [4]. As the core barrier to ecological security on the Qinghai-Tibet Plateau and even in China, the alpine grassland ecosystem provides several important ecological services, including water conservation, biodiversity protection, climate regulation, and support for local animal husbandry.

Since 1990, irrational human activities have gradually intensified and expanded, leading to the ongoing degradation of the grassland ecosystem in Qinghai Province [5]. At the same time, the combined effects of abnormal climate change, rodent infestation, and other natural factors have further worsened the condition of the grasslands, posing a serious threat to the stability of the province’s ecological environment and having far-reaching impacts on sustainable economic and social development [6]. Among these, grassland rodent infestation is a significant ecological issue that emerges during the development and utilization of grasslands. Over the years, the State has invested large sums of money in implementing highland grassland degradation control projects through various technical means, such as fence construction, sand control, grass planting, and so on. The ecology of the plateau has been greatly improved. However, many areas are still plagued by serious rodent infestation, which restricts the full recovery of grassland ecosystems. Relevant studies have shown that large areas of degraded grassland often become optimal habitats for rodents such as *Ochotona curzoniae* and *Eospalax baileyi* [7, 8]. The nibbling and frequent digging activities of these rodents have a serious impact on grassland vegetation and its substrate [9], creating a self-reinforcing vicious cycle. Consequently, rodent infestation has become a significant biological disaster that degrades the grassland’s ecological environment and hampers the sustainable development of animal husbandry. Therefore, it is particularly important to adopt scientific assessment methods to accurately define the degree of damage caused by rodents to grassland ecosystems. This not only helps to understand the occurrence and development trend of rodent damage but also provides a theoretical basis for the formulation of effective management strategies, thus achieving precise management and sustainable protection of grassland resources.

Traditional machine learning algorithms often face performance bottlenecks when dealing with large-scale data, making it difficult to effectively discover complex and nonlinear patterns in the data [10]. In contrast, deep learning techniques can address the challenges of large-scale data processing more efficiently with their powerful modeling capabilities and adaptive properties. In recent years, deep learning has been widely used in the field of ecological resources, covering such aspects as species monitoring and plant disease recognition. For example, Gomez *et al*. [11] utilized four deep convolutional neural networks—AlexNet [12], VGGNet [13], GoogLeNet [14], and ResNet [15]—to process and recognize wildlife images captured by cameras. Qiu *et al*. [16] employed Mask R-CNN [17] with ResNet50 as the backbone network to detect diseased regions in wheat. Mahum *et al*. [18] applied an enhanced DenseNet [19] to detect and classify potato leaf diseases. The above study highlights the broad potential of deep learning techniques in ecological resource monitoring and management.

However, convolutional architecture has limitations in dealing with global dependencies and long-range interactions, which are mainly constrained by local receptive fields, and it is difficult to adequately capture multi-scale features and global contextual information, thereby limiting their expressive ability in complex scenes. Building on the success of the Transformer [20] in natural language processing, Dosovitskiy *et al*. [21] explored its application in computer vision and proposed the Vision Transformer (ViT). Inspired by ViT, Zheng *et al*. [22] proposed the Segmentation Transformer (SETR), validating the feasibility and potential of the Transformer for semantic segmentation tasks. On this basis, semantic segmentation models based on the Transformer, such as Segmenter [23], SegFormer [24], MaskFormer [25], and Mask2Former [26], have continued to emerge. These models have made significant progress in improving segmentation performance and handling complex scenes.

This study aims to accurately identify and analyze rodent infestation in the plateau region by leveraging semantic segmentation technology in combination with the specific characteristics of rodent activity in Qinghai. The number of rodent burrows is a reliable indicator of rodent population density and serves as an important metric for assessing infestation levels. By applying semantic segmentation technology to images of rodent burrows in the plateau grasslands of Qinghai Province, this research calculates the proportion of rodent burrows in the local area, thereby inferring the extent of rodent-induced grassland degradation. SegFormer integrates a lightweight architecture with an efficient self-attention mechanism to ensure high accuracy while minimizing computational cost. In this study, we propose EM-SegFormer, an improved SegFormer model designed to more effectively capture subtle features and long-range dependencies in rodent burrow images. Particularly in scenarios with complex backgrounds and diverse terrains, the improved model demonstrates a significant enhancement in the accuracy of rodent burrow detection.The main contributions of this study are as follows:

1. We constructed a rodent burrows dataset from the highland grasslands of Qinghai Province, comprising 6,864 images (5,504 for training and 1,360 for testing), providing strong sample support for segmentation tasks.
2. We propose an improved rodent burrow segmentation model, EM-SegFormer, which incorporates the EMA module in the encoder to enhance global feature capture and cross-channel interactions. The MCFN is integrated into the decoder to improve feature fusion and detail recovery, enhancing segmentation accuracy.
3. Extensive experiments demonstrate that our model outperforms SegFormer and other mainstream models in rodent burrow segmentation task, and also exhibits strong generalization capability. Furthermore, significance analysis confirms that the improvements are statistically significant.

## Materials and methods

### Dataset description

Led by grassland workers, we conducted fieldwork in the highland areas of Qinghai Province. Using the DJI Air 3 drone (DJI, Shenzhen, China), we captured video footage from various times, locations, and viewpoints, gathering extensive data on rodent burrows. The videos were recorded in high clarity and prominently featured rodent burrows, providing valuable data for subsequent studies.

**Fig 1:**
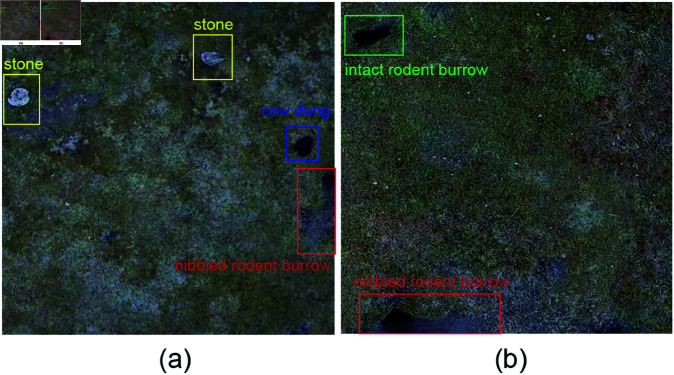
Sample images from the rodent burrows dataset. The instances in this figure are framed by solid red lines. From left to right in Fig 1(a), the four instances represent stone, stone, cow dung, and burrow1. In Fig 1(b), the two instances, from top to bottom, are burrow0 and burrow1.

**Fig 2:**
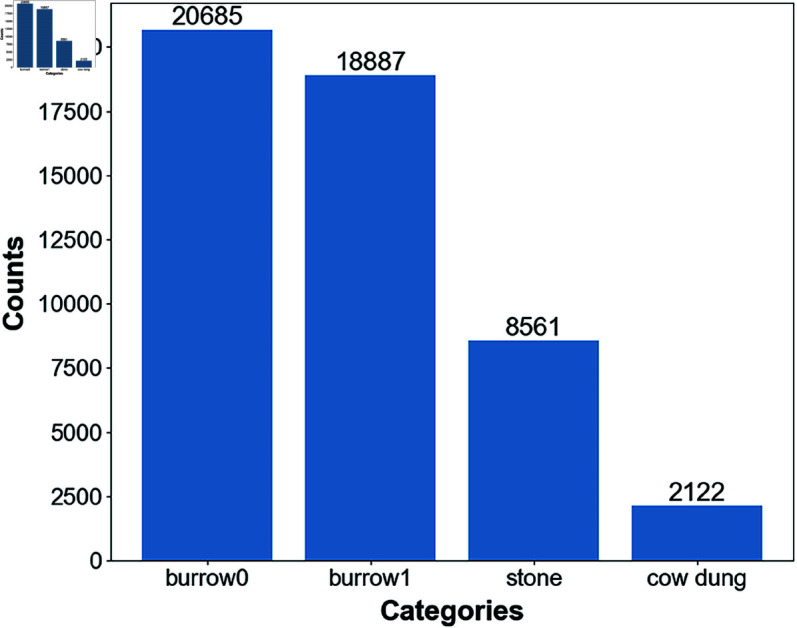
Dataset label distributition. The instance counts for the categories burrow0, burrow1, stone, and cow dung are 20,685, 18,887, 8,561, and 2,122, respectively.

In this study, OpenCV was used to extract frames from all the captured video files. Blurred and highly repetitive images were eliminated, resulting in a dataset of 6,864 valid rodent burrow images. The images were then labeled using the X-AnyLabeling tool, with a total of four categories: burrow0 (intact rodent burrow), burrow1 (nibbled rodent burrow), stone, and cow dung. The annotation work was carried out with the assistance of grassland workers, who have extensive experience in identifying rodent burrows. All the images were divided into a training set of 5,504 images and a validation set of 1,360 images at a ratio of 8:2, creating the rodent burrows dataset used in this study. A total of 50,255 instances were labeled in this dataset, each image contains at least one instance of the four categories. Fig 1 shows several sample images from the dataset, while Fig 2 illustrates the distribution of labels across each category. It can be observed that the edges of intact rodent burrows are relatively smooth, while the entrances of nibbled rodent burrows are damaged and exhibit signs of enlargement. Additionally, cow dung and rodent burrows look extremely similar in appearance, which adds some challenges to the improvement of the model.

### The EM-SegFormer model

With the successful application of ViT in computer vision, an increasing number of researchers have begun exploring the potential of Transformer models in semantic segmentation tasks. SegFormer is a simple, efficient, and powerful semantic segmentation model based on the Transformer architecture. By effectively combining the advantages of Transformer, SegFormer achieves excellent performance in various visual segmentation tasks.

SegFormer redesigns the encoder and decoder to create a hierarchical Transformer encoder that does not rely on positional encoding, along with a lightweight fully connected multilayer perceptron (MLP) decoder architecture. For different task requirements, the researchers designed a series of models, from SegFormer-B0 to SegFormer-B5, to meet the performance and efficiency needs of various scenarios. Among these, the SegFormer-B0 model has fewer parameters, making it suitable for resource-constrained environments, while the SegFormer-B5 model offers superior performance due to its larger capacity. In this study, given the limited computational resources, we opted to improve the SegFormer-B0 model to achieve efficient identification of rodent burrows while balancing performance and computational cost.

**Fig 3:**
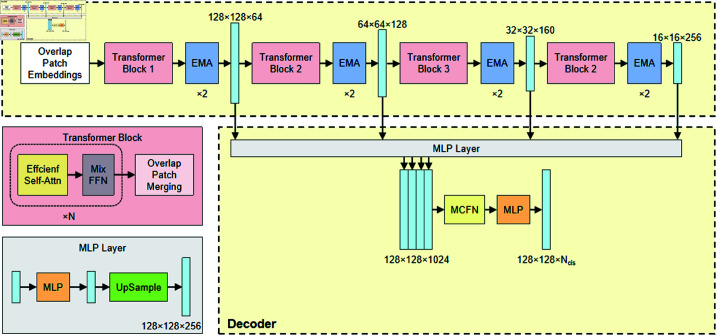
The EM-SegFormer framework.

In this paper, the encoder and decoder of the SegFormer-B0 model are each improved, with their structures shown in Fig 3.

The hierarchical Transformer encoder is employed to extract multi-level features from the image, including coarse features at high resolution and fine-grained features at low resolution. Each of the four stages in the Transformer Block stacks multiple Efficient Multi-head Self-Attention (EMSA) modules and a mixed feed-forward network. Unlike the traditional global attention mechanism, EMSA introduces local attention to efficiently implement multi-head attention, reducing computational complexity and memory consumption. However, this localized and efficient computational strategy may lead to insufficient information interaction between different channels. Given the small proportion of rodent burrows in the images and the subtle semantic and edge information, EMSA may struggle to adequately capture global information in tasks requiring precise global dependencies. To address these limitations, this paper integrates two EMA modules after the Transformer Block at each stage. This integration compensates for EMSA’s shortcomings in cross-channel information interaction and global information capture, enhancing the model’s performance in semantic segmentation tasks.

The encoder of SegFormer features a straightforward design, primarily consisting of MLP layers. The ALL-MLP decoder handles the fusion of multilevel features extracted by the encoder, and the specific process includes the following steps. First, the four feature maps are standardized to have the same number of channels through an MLP layer. Next, these feature maps are upsampled to match the resolution of the first feature map and concatenated. Then, the concatenated feature maps are further fused using another MLP layer. Finally, the fused features are processed through an additional MLP layer to produce the final predictive segmentation mask. This simplified design reduces the model’s computational complexity and parameter count, enhancing the network’s flexibility and efficiency. As a result, it is well-suited for a wide range of segmentation tasks. Nevertheless, it also comes with certain limitations. The ALL-MLP decoder may not be able to capture complex spatial context information through local receptive fields as effectively as convolutional decoders. It is also difficult to fully fuse this information when performing simple splicing operations on feature maps of different stages. Additionally, performing simple concatenation of feature maps from different stages makes it challenging to achieve comprehensive feature fusion.

### Efficient multi-scale attention mechanism

**Fig 4:**
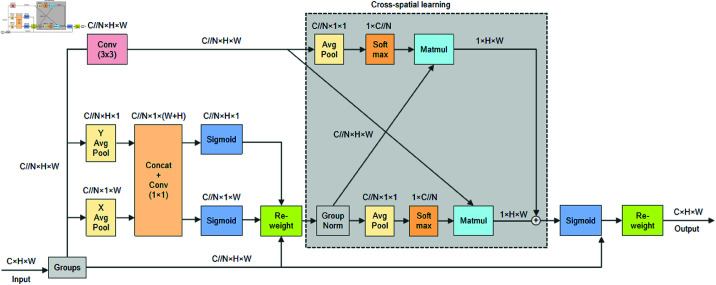
Illustration of EMA module.

In the field of computer vision, the attention mechanism plays an important role in improving model performance. By modeling the selective attention of the human visual system, the technique can efficiently focus on the most relevant features in the data, significantly enhancing both the efficiency and accuracy of the model. Coordinate attention [27] combines channel and spatial attention to optimize feature representation across both channel and spatial dimensions, enhancing the performance of downstream tasks such as image classification, object detection, and image segmentation. Yet, a process that models cross-channel relationships through channel dimensionality reduction essentially compresses the features, which may result in the loss of some information from the high-dimensional channels, thereby affecting the extraction of deeper feature representations. To address this, researchers have proposed the EMA [28] mechanism without dimensionality reduction, the structure of which is shown in Fig 4.

EMA divides the input feature map *X* into *N* groups of sub-features along the channel dimension, with each group learning different semantic features. Then it extracts the attention weights for the grouped feature maps through three parallel branches. On the one hand, in the two 1×1 branches, each has a 1D global average pooling operation to encode the channels in the horizontal and vertical directions. The encoded features are concatenated and passed through a 1×1 convolution without dimensionality reduction. The 1D global average pooling operations in the two directions can be represented by Eqs 1–2.

$$z_{c}^{H}(H)=\frac{1}{W}\sum_{0\leq i\leq W}x_{c}(H,i)$$ (1)

$$z_{c}^{W}(W)=\frac{1}{H}\sum_{0\leq i\leq H}x_{c}(j,W)$$ (2)

Where *H* and *W* represent the spatial dimensions of the input features, *c* denotes the number of input channels, and *x*_*c*_ refers to the input features of the *c*-th channel. The output of the 1 × 1 convolution is decomposed into two vectors, followed by the application of the Sigmoid function to generate a nonlinear mapping. Finally, the two channel attention maps are aggregated by multiplication. On the other hand, in the 3 × 3 branch, a 3 × 3 convolutional kernel is used to capture multi-scale feature representations. This cross-channel interaction effectively captures dependencies among all channels while preserving accurate spatial information, all with minimal computational cost.

EMA uses cross-spatial learning to aggregate feature information in the direction of different spatial dimensions. Specifically, the output of the 1 × 1 branch encodes global spatial information through 2D global average pooling, followed by a Softmax function. The formula for 2D global average pooling is presented in Eq 3.

$$z_{c}=\frac{1}{H\times W}\sum_{j}\sum_{i}x_{c}(i,j)$$ (3)

The output of the 3 × 3 branch is directly reshaped to the corresponding dimensions, and then the outputs of these two branches are aggregated through a matrix product to generate the first spatial attention map. In addition, a similar operation is applied to generate a second spatial attention map by performing 2D global average pooling on the outputs of the 3 × 3 branch, while the outputs of the 1 × 1 branch are directly reshaped to the corresponding dimensions. The outputs of the two branches are then aggregated using a matrix product. In the end, the output feature maps within each group are computed by aggregating the two spatial attention weight values and applying the Sigmoid function. The final output of the EMA has the same size as the input feature map *X*. EMA enhances cross-channel and cross-spatial feature interactions through finer feature aggregation and spatial attention, allowing the model to better extract global contextual information.

### Multi-kernel convolution feed-forward network

In the decoder architecture of SegFormer, the output feature maps from the four stages of the encoder are directly concatenated after upsampling. This strategy indeed enhances the model’s ability to capture multi-scale features. However, this approach also comes with potential issues: the feature maps from different stages may contain redundant information, and these redundant components could interfere with the model’s decision-making process. In addition, since these feature maps typically have different receptive fields and spatial resolutions, direct concatenation may lead to mismatched and lost information, which could negatively impact the overall performance of the model. To address the above issues, this paper innovatively introduces the MCFN [29] into the SegFormer decoder. The network effectively separates and extracts multi-scale feature information by performing convolutional operations at different scales, which reduces redundant content generated after feature splicing. At the same time, the MCFN also addresses the alignment problem between features at different scales, ensuring that feature maps from different encoder levels can be more accurately fused during the decoding process. This improvement enables the model to capture local details in the image more effectively, enhancing the recognition of small objects or intricate parts. This is particularly beneficial in tasks like segmenting rodent burrows in complex backgrounds, where accurately recognizing boundaries and fine details is crucial.

**Fig 5:**
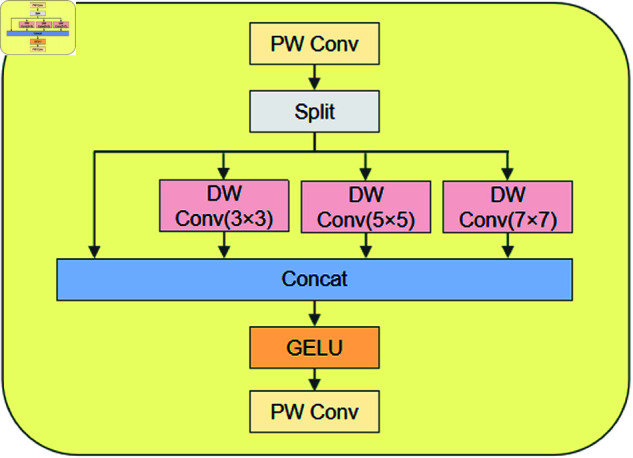
Illustration of MCFN.

As shown in Fig 5, MCFN first doubles the number of channels of the input features *F*_1_ using a pointwise convolution *f*_*pw*_. Next, a multi-kernel convolution operation *f*_*MC*_ is introduced, dividing it into four branches along the channel dimension. One branch retains the original information, while the other three branches employ a depthwise separable convolution with kernel sizes of 3 × 3, 5 × 5, and 7 × 7, respectively, to extract local information at different scales. Afterward, the feature maps from these four branches are concatenated. Nonlinearity is then introduced through a GELU function σ, and the number of channels is restored using a pointwise convolution operation to obtain the final output *F*_2_. The overall operation can be expressed by the following Eq 4.

$$F_{2}=f_{pw}\left(\sigma \left(f_{MC}\left(f_{pw}(F_{1})\right)\right)\right)$$ (4)

## Experimental design

### Experimental environment and parameters

This experiment is conducted on a Linux operating system with an Intel Xeon E5-2603 v4 CPU and an NVIDIA GeForce GTX 1080 Ti GPU. The deep learning framework used is PyTorch 1.10.1, with Python 3.8 and CUDA 11.3. The model is trained in a distributed manner using 8 GPUs, with a batch size set to 16. In the backbone, the overlapping patch embedding operation is configured with stride values of 4, 2, 2, and 2 across the four stages, respectively. Other training parameters are detailed in Table 1. To ensure the fairness of the experiments, all were conducted with consistent parameter settings, and each model was trained from scratch.

**Table 1:** Model training parameters.

Parameter	Value
Input image	512 × 512
Iteration	172000
Learning rate	0.0005
Optimizer	AdamW
Weight decay	0.01
Learning rate schedules	PolyLR

### Performance evaluation metrics

To objectively evaluate the model’s performance on the semantic segmentation task, we use evaluation metrics such as mean Intersection over Union (mIoU), precision, recall, and F1-score, to assess the model’s improvement. The formulas for these four evaluation metrics are shown in Eqs 5–8, where *TP* denotes the number of true positive samples, *FP* denotes the number of false positive samples, and *FN* denotes the number of false negative samples.

$$mIoU=\frac{1}{k+1}\sum_{i=0}^{k}\frac{TP}{FN+FP+TP}$$ (5)

$$Precision=\frac{TP}{TP+FP}$$ (6)

$$Recall=\frac{TP}{TP+FN}$$ (7)

$$F1=\frac{2\times Precision\times Recall}{Precision+Recall}$$ (8)

The mIoU measures the degree of overlap between the predicted and ground truth regions, comprehensively evaluating the model’s performance across all categories. It is a key metric for semantic segmentation. Precision evaluates how many of the samples predicted by the model as positive are indeed positive. At the same time, recall assesses how many of the actual positive samples are correctly identified by the model. The F1-score combines both precision and recall, with higher values generally indicating better model stability.

## Results and analysis

### Loss function curves

**Fig 6:**
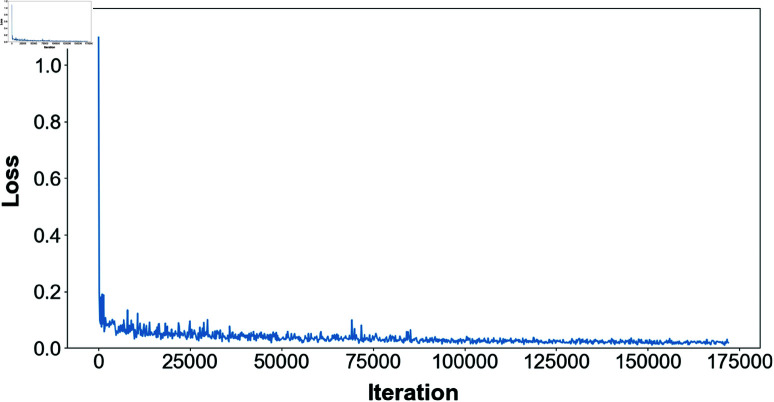
Loss function curve.

The loss function used by the model is the cross-entropy loss, which can effectively measure the classification error at each pixel point and is suitable for making independent category predictions at the pixel level, making it ideal for semantic segmentation tasks. Fig 6 illustrates the training loss variation of EM-SegFormer model on the rodent burrows dataset.

As shown in the figure, the loss value decreases rapidly in the early stages of training, suggesting that the model quickly learns basic features. As training progresses, the loss value gradually stabilizes, eventually fluctuating slightly around 0.02. This indicates that the model is undergoing fine-tuning to further optimize accuracy. However, the effect of these fine adjustments has saturated, signaling that the model has largely converged.

### Ablation study

We designed four sets of ablation experiments to evaluate the impact of different improvements on the performance of the baseline SegFormer model. According to the data analysis in Table 2, values are expressed as mean ± standard deviation (SD), calculated based on three independent sets of experimental data. The overall data distribution is more centralized and does not show wide fluctuations. When the EMA is introduced in the encoder, precision, recall, F1-score, and mIoU improve by 0.42%, 0.78%, 0.63%, and 0.86%, respectively. This improvement is attributed to the fact that the EMA module helps the model extract global contextual information more comprehensively through cross-channel and cross-spatial feature interactions. The MCFN employs multi-kernel convolutional operations to optimize the original ALL-MLP decoder in terms of multi-scale feature fusion and detail recovery. After integrating the MCFN into the decoder, the four metrics were improved by 0.17%, 2.01%, 1.16%, and 1.6%, respectively, leading to a marked enhancement of the model’s performance. Notably, the substantial increase in recall highlights that the improved model has strengthened its ability to identify rodent burrows in complex backgrounds, particularly in cases involving small or fuzzy rodent burrows.

**Table 2:** Enhancement of model metrics by different improvements.

Model	Precision	Recall	F1-score	mIoU
SegFormer	85.84 ± 0.18	80.86 ± 0.03	83.17 ± 0.09	72.40 ± 0.15
+EMA	86.26 ± 0.47	81.64 ± 0.32	83.80 ± 0.04	73.26 ± 0.06
+MCFN	86.01 ± 0.40	82.87 ± 0.33	84.33 ± 0.03	74.00 ± 0.03
EM-SegFormer	86.49 ± 0.42	83.36 ± 0.21	84.84 ± 0.10	74.68 ± 0.15

**Fig 7:**
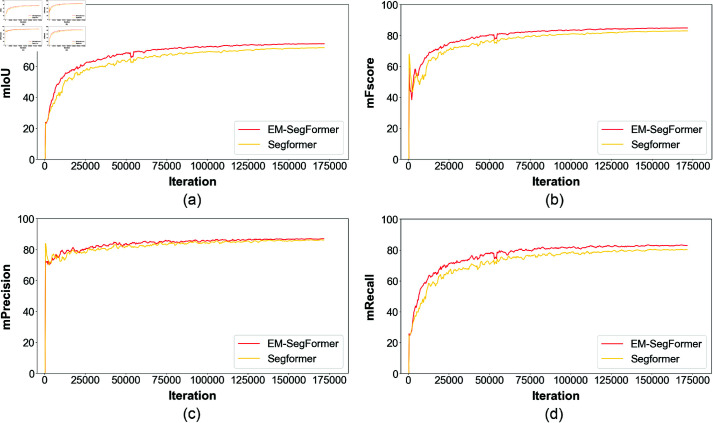
Performance metrics curve of SegFormer and EM-SegFormer. The sub panel (a) shows the mIoU change curve, (b) shows the F1-score change curve, (c) shows the precision change curve, and (d) shows the recall change curve.

**Fig 8:**
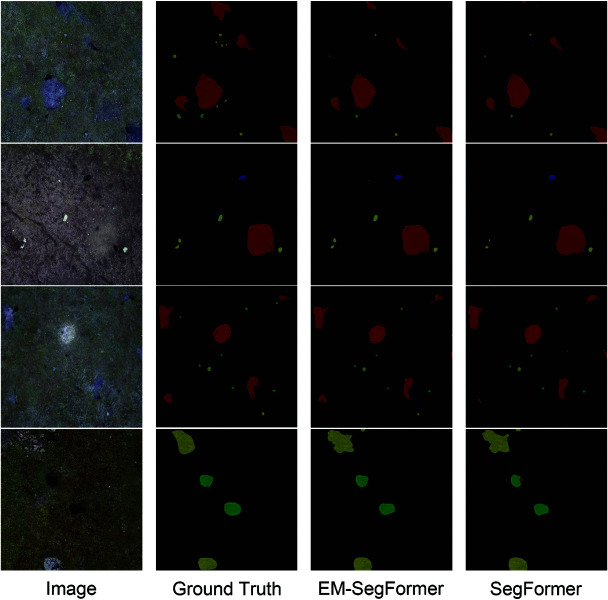
Comparison of prediction results between SegFormer and EM-SegFormer. Yellow represents stones, blue represents cow dung, red represents nibbled rodent burrows, and green represents intact rodent burrows.

Finally, our model improves mIoU from 72.40% to 74.68%, recall from 80.86% to 83.36%, F1-score from 83.17% to 84.84%, and precision from 85.84% to 86.49% on the rodent burrows dataset. The relatively small standard deviations indicate that the model exhibits stable performance across experiments, thereby supporting the reliability of the experimental results. The comparison curves of the performance metrics are shown in Fig 7. From the figure, we can visually observe that the performance of EM-SegFormer is comprehensively enhanced over the baseline SegFormer. As the number of iterations increases, the mIoU value gradually rises and stabilizes, suggesting continuous improvement in model performance. Similarly, the F1 score, Precision, and Recall values also increase, reflecting ongoing optimization in classification performance, positive sample classification, and positive sample identification, respectively. It indicates that the improved model has a lower leakage rate and relatively fewer misdetections in the prediction results, and can more accurately identify rodent burrows in the grass. Especially, the key metric mIoU is improved by 2.28%. This indicates that the optimized model has stronger feature extraction and fusion capabilities, which effectively improves the accuracy of rodent burrow segmentation, thus providing more reliable technical support for the assessment of rodent infestation situations. Fig 8 presents the prediction results of both models. Both models demonstrate effective segmentation results; however, SegFormer exhibits some misdetections, while EM-SegFormer produces slightly more accurate predictions.

### Significance testing

In order to verify whether the performance improvement of EM-SegFormer over SegFormer is attributable solely to random errors, we conducted significance tests on both models. First, we performed 5 experiments on each model using the rodent burrows dataset, yielding two sets of sample data. We focused on the mIoU, a key metric in semantic segmentation, and the relevant data are presented in Table 3. Multiple sets of experimental data show relatively stable results. Next, we applied an independent samples t-test to analyze the difference in the means of these two sample sets to determine whether any statistically significant difference exists.

**Table 3:** Results of model trials.

Experiment number	SegFormer mIoU	EM-SegFormer mIoU	Difference
1	72.23	74.75	2.52
2	72.50	74.51	2.01
3	72.46	74.78	2.32
4	72.49	74.81	2.32
5	72.46	75.04	2.58
Mean ± SD	72.43 ± 0.11	74.78 ± 0.19	2.35 ± 0.22

In the independent samples t-test, the t-value is a statistic that measures the difference between sample means. Larger t-values indicate a more substantial difference between the models, reflecting greater relative fluctuations and effects. On the other hand, p-values measure the probability that the observed difference is due to random fluctuations. Smaller p-values suggest that the difference between the new and old models is less likely to be attributed to random fluctuations. Typically, a p-value less than 0.05 is considered statistically significant.

Due to the complexity of manually calculating p-values, we typically rely on statistical software or libraries to perform these calculations. In Python, the scipy.stats library provides a comprehensive set of probability distributions and statistical functions, which we use to compute the t-value and p-value for the independent samples t-test. Based on the training results, we calculated *t* = 23.547 and *p* = 0.00002. This large t-value indicates that the difference in the mean mIoU values between the two sample groups is highly significant and well beyond the range of random fluctuations. Additionally, the p-value is much smaller than the commonly used significance level of 0.05 and even smaller than 0.01, providing strong evidence that the observed mean differences are statistically significant and not due to chance.

With this significance test, we confirm that the improvement in performance of EM-SegFormer is reliable and that the improvement is statistically significant, further validating the effectiveness of our model improvement.

### Comparative experiment

To further illustrate the model’s excellent performance in segmenting rodent burrows, we also selected several other mature semantic segmentation models to compare with EM-SegFormer. The experimental results, as shown in Table 4, indicate that EM-SegFormer achieves the highest mIoU on the rodent burrows dataset, despite utilizing the fewest parameters.

**Table 4:** Performance of different models on the rodent burrows dataset.

Model	Precision	Recall	F1-score	mIoU	Params(M)
UNet [30]	86.16	82.98	84.46	74.15	28.99
DeepLabV3+ [31]	87.27	82.62	84.82	74.66	12.32
PIDNet [32]	85.87	81.39	83.45	72.83	37.31
SegNeXt [33]	86.28	81.78	83.88	73.39	13.89
PoolFormer [34]	86.30	80.04	82.97	72.08	34.60
EM-SegFormer	86.61	83.30	84.87	74.75	7.96

Specifically, EM-SegFormer records the highest mIoU (74.75%) and F1-score (84.87%), slightly surpassing DeepLabV3+ in these metrics, while also achieving the highest recall (83.30%), highlighting its capability to identify true positives more effectively. Although the precision of EM-SegFormer is marginally lower than DeepLabV3+, it remains competitive. Compared to the classical UNet, our approach improves on all performance metrics. Notably, EM-SegFormer achieves this superior performance with the fewest parameters, significantly less than models such as PIDNet and PoolFormer. Taken together, our proposed method is suitable for application in resource-constrained environments. Its excellent overall performance demonstrates the effectiveness of our improvements and innovations in model design, offering a strong solution for rodent infestation assessment.

### Generalization evaluation

In this study, the Cityscapes dataset is introduced to evaluate the generalization ability of the proposed model under varying data distributions. This dataset comprehensively captures the complexity of real city street environments and includes video sequences from 50 different city streets [35]. A total of 5000 images are provided with high-quality pixel-level annotations, which consist of 2975 training images, 500 validation images, and 1525 test images, covering 19 semantic categories.

The applicability of the model to other complex scenarios is validated through experiments conducted on the Cityscapes dataset. Specifically, three sets of experiments were carried out on this dataset using EM-SegFormer and SegFormer, respectively. The results of these experiments are similarly summarized in Table 5, presented as mean ± SD. The experimental results demonstrate that the proposed model achieves varying degrees of improvement across multiple evaluation metrics compared to the benchmark model, with lower result fluctuations and more stable performance gains. In particular, the mIoU increases from 72.82% to 74.41%, further indicating that the model exhibits excellent semantic segmentation performance in urban street scenes. These findings highlight the model’s strong generalization ability and robustness, thereby confirming its broad applicability and potential for real-world deployment.

**Table 5:** Evaluation results on the Cityscapes dataset using EM-SegFormer and SegFormer.

Model	Precision	Recall	F1-score	mIoU
SegFormer	86.27 ± 0.29	81.12 ± 0.32	83.43 ± 0.09	72.82 ± 0.11
EM-SegFormer	86.74 ± 0.23	82.70 ± 0.15	84.52 ± 0.11	74.41 ± 0.13

## Discussion

The traditional method of counting rodent burrows relies on manual field surveys, which are time-consuming, labor-intensive, and ineffective for accurately monitoring rodent infestations across vast grasslands due to their large area and widespread rodent populations. The drone hyperspectral technology improves detection accuracy and reduces labor costs [36, 37], but remains costly, complex in data processing, and sensitive to environmental factors, with limited performance in detecting small targets and recognizing shapes. In this study, our proposed semantic segmentation method based on the SegFormer model shows good performance in rodent infestation monitoring in highland areas. Compared to other common image segmentation methods, such as UNet, Mask2Former, PoolFormer, and others, our model has significant advantages in handling segmentation tasks with small targets and complex backgrounds. UNet often faces the problem of degradation of segmentation accuracy when dealing with images with small targets [38]. Mask2Former has relatively high computational complexity, resulting in longer training times and greater resource consumption, making it unsuitable for use under our available conditions. PoolFormer, as a lightweight architecture, utilizes pooling operations instead of complex attention mechanisms, but pooling operations may lead to the loss of small-scale features. Our experimental results also show that PoolFormer is not as effective as EM-SegFormer in the plateau rodent infestation monitoring task.

Although EM-SegFormer has good performance and keeps the number of parameters low, the improvements introduced add some computational cost. According to the experimental log, the training time of the original SegFormer model was 11 hours, while the training time of the improved model increased to 20 hours. Despite this increase, the additional training time does not significantly affect real-world applications, as the training process is performed only once. Furthermore, the improved model’s enhanced performance leads to better results during the subsequent inference phase. During inference, we tested 1360 images with the total inference time recorded at 103.84 seconds, averaging 0.0764 seconds per image, and an average memory usage of 1648.81 MB. These results demonstrate that the efficiency and resource consumption of the improved model in the inference phase remain within acceptable limits, confirming its feasibility for practical applications.

The method proposed in this study aims to more effectively detect the relationships among the behavior, population dynamics, and ecological impacts of plateau rodents through an intelligent approach. It offers strong technical support for ecological and environmental research and is particularly well-suited for application in high-altitude regions. By providing automated monitoring tools, the method is expected to substantially reduce the physical burden and visual fatigue experienced by grassland workers operating under harsh environmental conditions, thereby enhancing operational efficiency and safety. Nevertheless, the deployment of such technologies must prioritize animal welfare to ensure that target species and their habitats are not disturbed or harmed. In certain scenarios, it is also necessary to mitigate the negative ecological impacts of rodent activity through restoration measures such as artificial grass planting and the installation of protective fencing. Moreover, data collection and model development should strictly adhere to relevant ethical guidelines to avoid misuse in inappropriate contexts and to prevent potential ecological risks [39].

Future research should further consider the environmental and social consequences that may arise during the dissemination of this technology, and actively promote the responsible and standardized application of artificial intelligence in ecological conservation. For instance, combining artificial vegetation restoration with intelligent monitoring can help protect plant life while maintaining plateau rodent populations, thus supporting biodiversity and ecological balance. We will also continue to explore lightweight design schemes such as distillation learning [40], network pruning [41], and other methods to further reduce the computational complexity. We plan to continuously expand the dataset to enhance the generalization ability of the model and improve its adaptability in real-world scenarios, while fully leveraging the advantages of the Transformer architecture.

## Conclusion

In this study, semantic segmentation was utilized to monitor and evaluate the rodent infestation in highland grasslands. Through field research, we constructed a dataset covering the information of rat holes, and introduced the EMA mechanism and MCFN module into SegFormer to enhance the segmentation ability of the model in complex backgrounds. The experimental results show that the improved EM-SegFormer model achieves significant improvement in segmentation accuracy, which further validates its superiority. The research results not only provide a more accurate technical means for monitoring rodent infestation in the plateau, but also lay a foundation for the application of semantic segmentation technology in the field of ecological resources. In the future, our research will continue to optimize the performance of the model, promote the development of intelligent monitoring technology, and provide more reliable technical support for the accurate assessment and efficient management of ecological environment.
